# Legislation—Pennsylvania

**Published:** 1895-03

**Authors:** 


### LEGISLATION.

### Pennsylvania.

*House Bill No.* 365. *An Act Legalizing the Dehorning of Cattle.*

Section 1. *Be it enacted by the Senate and House of Representatives of the Commonwealth of Pennsylvania in General Assembly met, and it is hereby enacted by the authority of the same*, That from and after the passage of this act it shall be lawful for any person or persons of this Commonwealth to dehorn cattle.

*House Bill No.* 308. *An Act to Prevent the Nicking, Docking and Mutilation of Horses' Tails.*

Section 1. *Be it enacted by the Senate and House of Representatives of the Commonwealth of Pennsylvania in General Assembly met, and it is hereby enacted by the authority of the same*, That from and after the passage of this act it shall be unlawful for any person or persons to either nick, dock, or mutilate the tail of any filly, colt, mare, gelding or stallion: *Provided*, That this act shall not be construed to apply to the scientific straightening of horses' tails.

Section 2. Any person violating the provisions of the first section of this act shall be guilty of a misdemeanor, and upon conviction thereof shall be fined by the court of the proper county not to exceed fifty dollars for each offense.

